# Sibling Involvement and Documentation in Pediatric Eating Disorder Care

**DOI:** 10.1002/eat.70072

**Published:** 2026-03-24

**Authors:** Amalie Schumann, Elizabeth Rapa, Louise Dalton

**Affiliations:** ^1^ Department of Psychiatry University of Oxford Oxford UK

**Keywords:** eating disorders, family care, family‐based treatment, health records, NICE guidelines, siblings

## Abstract

**Objective:**

When a young person has an eating disorder (ED), the entire family, including siblings, is affected. Despite recommendations in clinical guidelines and treatment manuals to involve and support siblings, little is known about how this is enacted and documented in practice. The aims of this study were: (1) to audit documentation of sibling information and involvement in child and adolescent ED services and its alignment with clinical guidelines, and (2) to explore healthcare professionals' perspectives on sibling involvement.

**Method:**

A mixed‐methods design was used. Clinical records of 102 young people receiving ED treatment were reviewed, and data were extracted using a data collection tool. Data were analyzed descriptively. A focus group discussion was conducted with 14 healthcare professionals. Data were analyzed qualitatively, applying thematic analysis.

**Results:**

Eighty‐six of 102 patients (84%) had siblings (*n* = 160 siblings across the sample). Attendance in sessions was documented for 20 of the 160 siblings (13%) across 19 of the 102 patients (19%). Across all records, one record documented sibling perspective and support offered. Qualitative analysis of the focus group identified six themes. Sibling involvement was seen as overwhelming, constrained by workload, privacy concerns, and lack of appropriate resources, and conceptualization of involvement was limited to session attendance. Perceived benefits included enhanced systemic insight and improved family communication.

**Discussion:**

In this study, documentation of sibling involvement in child and adolescent ED care was limited. Clearer documentation practices, broader conceptualizations of sibling involvement, and further research are needed to ensure siblings receive appropriate support.

## Summary

- Sibling involvement in child and adolescent eating disorder services was limited, despite clinical guidelines and treatment manuals recommending sibling inclusion and support.
- Clinicians described barriers to sibling involvement including workload, service and family logistics, and privacy concerns, but also recognized benefits and opportunities for sibling inclusion.
- Broader conceptualizations of sibling involvement, clearer documentation practices, and better resources are needed to ensure siblings are informed and supported.

## 1 Introduction

When a child develops an eating disorder (ED) the whole family is affected. However, research indicates that siblings' experiences and needs are often overlooked (Persico et al. 2021; Scutt et al. 2022), and siblings are at risk of developing psychological difficulties themselves (Heneghan et al. 2024). Across qualitative studies, siblings report that EDs are difficult to understand, express a need for information about the diagnosis, better support, and greater involvement in their ill sibling's care (Fjermestad et al. 2020; Jungbauer et al. 2015; van Langenberg et al. 2018; Schumann et al. 2025).

Family‐based approaches are the first‐line treatment for children and young people with anorexia nervosa (AN) and bulimia nervosa (BN), consistently supported by international clinical guidelines including those for the United Kingdom, United States, and Canada (American Psychiatric Association 2023; Couturier et al. 2020; National Institute for Health and Care Excellence (NICE) 2020). In England, The National Institute for Health and Care Excellence (NICE) guidelines are the official standards expected to be followed. Guideline 69 (NG69) outlines recommendations for the recognition and treatment of EDs, explicitly including siblings in the definition of “family members” (NICE 2020).

Regardless of the patient's age, diagnosis, or treatment type, the guideline advises clinicians to “*find out what the patient and their family members know about the eating disorder and address any misconceptions*” (NICE 2020, NG69, 1.1.3), and to “*be aware that family members may feel guilty or responsible for the eating disorder*” (NICE 2020, NG69, 1.1.4). Specifically for children and young people (< 18 years) with AN or BN, the guideline recommends offering family therapy (FT‐AN and FT‐BN) as the first‐line treatment (Eisler et al. 2016; Le Grange and Lock 2007; NICE 2020). In both cases, treatment should also include “*support for family members who are not involved in the family therapy, to help them cope with distress caused by the condition*” (NICE 2020, NG69, 1.3.12 and 1.5.8).

Family‐based approaches for treatment of EDs are grounded in the theory that successful recovery requires the active involvement of family members, including siblings (Eisler et al. 2016; Le Grange and Lock 2007; Lock and Le Grange 2025). Two studies have explored sibling participation in family‐based treatment approaches, with both finding that sibling involvement was minimal (Gustafsson et al. 1995; Hughes et al. 2018). Siblings of patients with AN attending an outpatient clinic in Australia had the lowest participation of family members, with a mean attendance rate of 20% (Hughes et al. 2018). Approximately one‐quarter (26%) of the siblings did not attend any sessions, and only 1% attended all the sessions (Hughes et al. 2018). A study with siblings of Child and Adolescent Mental Health (CAMHS) outpatients in Sweden found that almost 40% of siblings did not participate in any family therapy sessions, and only 14% attended three or more (Gustafsson et al. 1995). However, the current relevance of this study must be interpreted with caution due to potential changes in practice since the study was undertaken.

Despite recommendations to involve, inform, and support siblings, there is limited evidence on how, and to what extent, NICE guidelines are implemented in practice, how sibling involvement is documented, and clinicians' attitudes toward sibling involvement.

## 2 Objectives

1. To audit the documentation of sibling information and involvement in CAMHS ED care, and assess whether clinical records documented attention to siblings in line with NICE guidelines.
2. To explore healthcare professionals' (HCPs) perspectives on the results of the audit and the benefits, challenges, and practicalities of sibling involvement.

## 3 Method

### 3.1 Setting and Design

Data were collected in a CAMHS ED service in England. A mixed‐method design was used to provide (1) a structured review of health records of young people receiving ED treatment at a single time point; and (2) a qualitative understanding of HCPs' attitudes to inform interpretation of quantitative data and HCPs' perceived benefits, challenges, and opportunities to sibling involvement.

### 3.2 Data Collection Procedures

#### 3.2.1 Review of Health Records

Records were reviewed for patients currently receiving treatment in the service on July 31, 2024. Data extraction from the patient records of those identified took place from August to October 2024. A data recording tool using Excel and a search strategy were developed by the first author in collaboration with clinicians working in the service to ensure consistency in data recording. Demographic sheets in the clinical records were examined, and the entirety of each record was searched using a keyword list (variants of sibling, family members, and parents; see Appendix A for the full list). The notes around each “hit” were reviewed and relevant data recorded.

Patient's year of birth, sex assigned at birth, number of mental health services involved, current type of treatment, number of inpatient admissions, and number of siblings were recorded. Sibling age, sex assigned at birth, presence in any consultations and number of consultations, information recorded about reasons for sibling (non) involvement, and any additional notes related to siblings were recorded.

#### 3.2.2 Focus Group With HCPs

An in‐person focus group was conducted with 14 HCPs working in the service in February 2025, facilitated by two of the researchers (AS and LD). A semi‐structured topic guide (Appendix B) was developed to explore HCPs' reflections on the audit results, the extent to which these resonated with their perceived practice, and attitudes toward sibling involvement. Participants were invited to write their ideas on Post‐it notes before a wider group discussion. The notes were collected and transcribed as part of the data material. The group discussion was audio recorded and transcribed, and lasted 81 min.

### 3.3 Eligibility Criteria

Patients currently receiving treatment (July 31, 2024) were eligible for inclusion. Records for patients awaiting assessment or on a waiting list for assessment were excluded. A total of 110 patients were eligible for inclusion. HCPs employed by the ED service were invited to take part in the focus group.

### 3.4 Data Analysis

Health record data was analyzed using descriptive statistics. Central tendency for continuous variables was reported using means, and variability described by standard deviations and range. Categorical variables were presented as percentages and counts, and missing data were displayed.

To explore if clinical records captured attention to the impact on siblings and information they had received (NG69 1.1.3), or support offered to siblings (NG69, 1.3.12/1.5.8), the first author coded the data for each patient, and consulted the findings with senior members of the research team and the clinical team to ensure consensus. The focus group discussion was audio recorded and transcribed verbatim by the first author. The transcript was analyzed using thematic analysis, following the six‐step framework outlined by Braun and Clarke (2006, 2019): (1) familiarization with the data; (2) generating initial codes; and (3–5) searching for, reviewing, and defining themes (Braun and Clarke 2006, 2019). Participant validation (Lincoln and Guba 1985) was conducted with two focus group participants who read and commented on a draft of the manuscript, including the results and discussion. They reported that the findings reflected their experiences. Public and Patient Involvement (PPI) contributors were consulted during manuscript preparation and indicated that the findings resonated with their experiences.

### 3.5 The Involvement of Persons With Lived Experiences

There is established Patient and Public Involvement (PPI) oversight of the research group's work on supporting siblings of young people with mental illness including parents and siblings, and clinicians working in ED services. For the current study, PPI was consulted for the manuscript.

## 4 Ethics

The project was registered and approved as a clinical audit project by an NHS Foundation Trust (CA/2024‐25/01). HCPs consented to the focus group being recorded and transcribed, and to the publication of their anonymized quotes and contributions.

## 5 Results

### 5.1 Health Records Review Results

The patient and sibling characteristics and documentation of sibling involvement are presented in Table 1. A total of 102 patients receiving treatment for a clinically confirmed ED were included, representing 93% (102/110) of all eligible patients. Eight records were not reviewed due to changes in the clinical team, which resulted in resource constraints. Most of the patients (84%; 86/102) were recorded as having siblings, with a total of 160 siblings identified. Sibling attendance in one or more consultations was documented for 13% of siblings (20/160), corresponding to 19% (19/102) of patients. Family‐based treatment approaches were recorded for 45% (46/102) of patients. Among these patients, 83% (38/46) had siblings, and sibling participation in at least one session was documented in 26% (10/38) of cases.

**TABLE 1:** Patient and sibling characteristics, and documentation of sibling involvement.

Variables	Results
Patient characteristics	
Sample size, *n*	102
Age, mean (SD), range, years	15.4 (1.9) 8–18
Sex assigned at birth, *n* (%)	
Female	95 (93%)
Male	7 (7%)
Patients with ≥ 1 inpatient admissions, *n* (%)	15 (15%)
Inpatient admissions, mean (SD), range	0.2 (0.6) 0–4
Missing, *n*	4
Clinical teams involved, mean (SD), range	1.9 (1.1) 1–6
Missing, *n*	5
Sibling characteristics	
Patients who have siblings, *n* (%)	86 (84%)
Missing, *n*	1
Siblings identified across all patients, *n*	160
Missing (patients where sibling count not reported), *n*	4
Sibling age, mean (SD), range, years	13.1 (5.0) 2–23
Siblings under 18 years old, *n* (%)	75 (47%)
Missing, *n* (% of total sibling sample)	42 (26%)
Sibling sex assigned at birth, *n* (%)	
Female	73 (46%)
Male	39 (24%)
Missing	48 (30%)
Documentation of sibling attendance in consultations	
Of total sibling sample: siblings present in ≥ 1 consultation(s), *n* (%)	20 (13%)
Of total patient sample: patients where siblings have been present in ≥ 1 consultation(s), *n* (%)	19 (19%)
Among siblings who attended consultation(s): consultations attended, mean, (SD), range	2.8, (2.9), 1–11
Documentation of sibling attendance in family‐based treatment approaches	
Patients documented to currently be receiving family‐based treatment, *n* (%)	46 (45%)
Of those patients receiving family‐based treatment: patients with sibling(s), *n* (%)	38 (83%)
Of those patients who have sibling(s) and receive family‐based treatment: patients where a sibling had been present in ≥ 1 consultation(s), *n* (%)	10 (26%)
Among siblings who attended family‐based session(s): consultations attended, mean, (SD), range	3.4, (3.4), 1–11

One set of clinical notes captured attention to NG69 1.1.3 (documenting the ED's impact on the sibling, and information the sibling had received from the clinical team). For siblings not involved in family‐based treatment approaches, no records captured attention to NG69, 1.3.12/1.5.8 (documenting support offered or provided to help family members cope with distress caused by the condition).

### 5.2 Qualitative Results

In the focus group discussion, HCPs noted that some sibling information may not be documented due to privacy concerns, reflecting that they “*sometimes knew more about siblings than records show*” (e.g., sibling age). However, most HCPs reported that the audit findings reflected their perception of siblings' involvement in patient care, with limited inclusion and multiple barriers to involvement. Six themes were identified, and the themes and supporting quotes are presented in Table 2.

**TABLE 2:** Qualitative themes: HCPs' reflections on sibling involvement.

Name and description of themes	Supporting quotes
Theme 1: Competing priorities	
Sibling involvement was perceived as overwhelming for HCPs, particularly when they were faced with already high workloads. HCPs also experienced parents to be overwhelmed by sibling attendance. Perceived priority was the patient who was often at high risk, or with involving parents rather than siblings.	*“We've got cases that keep us quite busy. Is there space, mental space, to think about them [siblings] as well?” (Participant 2)* *“You sort of almost choose which battle to fight, to prioritise both parents being there and instead of you know, being able to accommodate for the siblings” (Participant 6)* *“More children to worry about in the session [for the parent]” (Anonymous note)*
Theme 2: A desire to shield siblings	
Attendance was perceived as potentially distressing for both the sibling and the patient. Siblings were considered to need shielding from certain topics, such as suicide risk.	*“[parents are] worried the sibling will copy the ill child” (Anonymous note)* *“Some things are not appropriate for a sibling to hear, especially if sibling is young, for example suicide risk” (Anonymous note)* *“Trauma‐exposure and too much distress in the treatment setting for the sibling” (Anonymous note)*
Theme 3: Concerns about disruption and limited benefit	
Potential value of sibling involvement dependent on the sibling's age and the age gap with the patient. Sibling attendance could sometimes be unhelpful for both the patient and the sibling. For example, younger siblings might struggle to understand the situation. Siblings could disrupt the session, for example, by dividing the parents' attention.	*“I think in some sessions that I've observed having the sibling there and the sibling being younger has often been unhelpful for the patient […] the young brother would make comments that would be unhelpful to the situation in general, but also just considering the age of the younger brother, there was not that maturity or that understanding of what was actually going on” (Participant 10)* *“When the parents are there with a buggy with them, then you just know you're not gonna get the parents' attention. They're going to be split” (Participant 4)*
Theme 4: Practical and logistical challenges	
Lack of space and rooms, and difficulties with scheduling appointments. Challenging for parents to manage the logistics of bringing siblings along.	*“I think a lot of the building setup doesn't accommodate for siblings” (Participant 3)* *“Lack of space to see the whole family and time to give everyone a voice” (Anonymous note)* *“Difficult to find appointments that suit everyone” (Anonymous note)* *“Both children go to different schools and the parents have to manage work/life” (Anonymous note)*
Theme 5: Privacy concerns and secrecy impact sibling involvement	
Perceived as inappropriate to record information about siblings in the patient's health records. Parents and patients might be less honest if siblings are present due to feelings of shame and guilt. Secrecy around the ED within the family and wider network can shape how siblings understand the diagnosis.	*“Sibling involvement might feel like a breach of privacy for the young person” (Anonymous note)* *“It struck me just then thinking I know one of the narratives that families hold with their siblings is to say they have a condition, a heart condition, and that's what they might tell school as well. So then siblings are misinformed because they think, oh my sibling's got a heart condition” (Participant 7)* *“Feelings of shame and jealousy – unable to express this if sibling is present (from the young person point of view)” (Anonymous note)* *“Parents might be worried about speaking openly about what is not going well” (Anonymous note)*
Theme 6: Conceptualizing sibling involvement	
Sub‐theme 6.1 Siblings brought to sessions	
HCPs' perceptions of how siblings could be involved in treatment were largely limited to their attendance in appointments with parents and patients. Bringing siblings to consultations was inconsistent and not offered as part of routine care.	*“If parents have talked about it, we've tried to be creative. [Sibling attendance] is something we could offer ones or twice off. But we've never had a formal process. Yeah, I think it's always been very ad hoc” (Participant 2)*
Sub‐theme 6.2: Perceived benefits	
Siblings offer unique insights into the family system, supporting more systemic and holistic thinking. Siblings could provide a sense of normality and play a helpful role in the treatment process and recovery. Brings the whole family together: a space for the patient and the sibling to talk honestly.	*“The sibling can say things the young person is scared of saying” (Anonymous note)* *“Siblings support normal family time outside of appointments” (Anonymous note)* *“Sibling involvement allows the HCP to think systemically and holistically” (Anonymous note)* *“The sister was 10 and she's 15. Coming back was really useful. This isn't something we would wanna do every week. We've had two sessions like that and it gave both siblings space to talk about what they're worried about” (Participant 11)* *“But some of those families where the entire family have come, you've really got to know the whole family, including the siblings, it feels really good when you do have that.” (Participant 3)*
Sub‐theme 6.3: Lack of resources and appropriate services	
HCPs were concerned that siblings would need additional support and that appropriate services did not exist for any onward referrals. Perception that HCPs did not have suitable resources and materials that could be shared with siblings.	*“If you identify a mental health need, there's no service to access.” (Anonymous note)* *“The difficulty I've found is when trying to find age‐appropriate resources to share, particularly when you've got siblings of a range of ages. And knowing what information to share with which one and how to do it” (Participant 13)*

## 6 Discussion

This study describes the documentation of sibling information and involvement in CAMHS ED services and HCPs' perceptions of sibling involvement.

The review of patient records showed limited documentation of sibling involvement, and the focus group provided insight into multiple barriers of sibling engagement. Contrary to this, clinical recommendations and treatment manuals emphasize the importance of considering sibling inclusion and support (Eisler et al. 2016; Le Grange and Lock 2007; NICE 2020). The findings are consistent with the few existing studies on sibling engagement, reporting limited involvement in family‐based treatment approaches (Gustafsson et al. 1995; Hughes et al. 2018) and reflect siblings' reports of isolation and exclusion from ED care, despite their wish for more information and involvement (Fjermestad et al. 2020; Jungbauer et al. 2015; van Langenberg et al. 2018).

In the focus groups, HCPs described multiple reasons for the limited sibling engagement, such as high workloads and feeling overwhelmed, complex cases with high risk, several practical and logistical challenges to sibling involvement, and a lack of appropriate resources and services to refer siblings to. HCPs' feelings of being overwhelmed and unable to priorities siblings are consistent with wider literature (Novogrudsky et al. 2025). When workload pressures are high, it becomes increasingly difficult for services to go beyond immediate patient needs (Novogrudsky et al. 2025).

HCPs perceived that their own desire to protect siblings from aspects of the ED was shared by both parents and patients. This contrasts with siblings' own reports, which emphasize a desire for greater involvement and information during their brother or sister's ED treatment (Jungbauer et al. 2015; Nilsen et al. 2021; van Langenberg et al. 2018).

HCPs principally viewed sibling involvement as attendance in sessions. However, sibling involvement can be conceptualized more broadly to include, for example, guiding parents on how to support siblings or holding individual conversations with siblings themselves. Qualitative studies highlight siblings' desire for independent support, peer support, and direct information about the ED from parents or the clinical team (Batchelor et al. 2022; Jungbauer et al. 2015).

## 7 Limitations

This study was conducted within a single service in England, limiting the generalizability of findings. The qualitative data may have been influenced by the large group size, group dynamics or the dominance of particular voices. To mitigate this, participants were invited to write down anonymized reflections, aiming to capture a range of perspectives, including from those less likely to speak up in the group. The researchers were not part of the clinical team working in the service but have ongoing collaborations. Discussing experiences with individuals they already knew may have influenced what participants chose to share.

The audit relied on clinical records, and HCPs may have explored sibling involvement or discussed sibling support without documenting this. This was addressed by encouraging HCPs to feedback and comment on the audit results during the focus group. Another limitation of this study is that service users or individuals with lived experience were not involved in the development of the topic guide.

## 8 Implications for Research and Practice

Future studies should adopt multi‐site designs to better understand current approaches to sibling involvement in ED services nationally and internationally. Clinically, there is a need for greater clarity on where and how to document sibling information and involvement so that it remains accessible to HCPs working with the family whilst also complying with data protection and confidentiality.

Training should encourage broader conceptualizations of sibling involvement, beyond sibling attendance in sessions, such as through psychoeducation, guided parent support, or individual work with siblings where appropriate. Addressing these issues may require more resources in services, further research involving siblings, parents and patients, and development of specific sibling interventions.

## 9 Conclusion

This study highlights limited sibling engagement in CAMHS ED services, despite recommendations in NICE guidelines and treatment manuals. HCPs reported barriers including workload pressures, privacy concerns, lack of appropriate resources, and family and service logistics. Strengthening documentation practices, expanding approaches beyond session attendance, and providing resources and clearer guidance may improve sibling support and inclusion.

## Author Contributions

**Amalie Schumann:** conceptualization, methodology, formal analysis, data curation, project administration, writing – original draft, writing – review and editing. **Elizabeth Rapa:** conceptualization, methodology, writing – review and editing, supervision. **Louise Dalton:** conceptualization, methodology, project administration, writing – review and editing, formal analysis, supervision.

## Lived Experience Involvement Statement

There is established Patient and Public Involvement (PPI) oversight of the research group's work on supporting siblings of young people with mental illness including parents and siblings, and clinicians working in eating disorder services. For the current study, PPI was consulted for the manuscript.

## Funding

The first author receives funding from the Aker Scholarship.

## Ethics Statement

The study was registered and approved by an NHS Foundation Trust (name redacted) as a clinical audit project. Registration code: CA/2024‐25/01.

## Consent

The authors have nothing to report.

## Conflicts of Interest

The authors declare no conflicts of interest.

## Supporting information


**Appendix A** List of search terms for review of patient health records.
**Appendix B** Focus group topic guide.

## Data Availability

The data supporting this study's findings are not publicly available but may be shared upon reasonable request. Data are available from the author(s) with the permission of the NHS Foundation Trust. Data are located in controlled access data storage at the University of Oxford and the NHS Foundation Trust.
