# Supplementary material for: Sibling Involvement and Documentation in Pediatric Eating Disorder Care
Source: Int J Eat Disord. 2026 Mar 24;59(7):1635–42. doi: 10.1002/eat.70072 (PMC13326767; doi:10.1002/eat.70072)
Supplement: Supplementary file 1 — Appendix A List of search terms for review of patient health records. Appendix B Focus group topic guide. [file EAT-59-1635-s001.docx]

**Appendices**

**Appendix A: List of search terms for review of patient health records.**

| **Search terms** |
| --- |
| Sibling / sister / brother / twin / [name of sibling if known] |
| Parent / carer / caregiver / guardian |
| Mum / mother / [name of mother] |
| Dad / father / [name of father] |

**Appendix B: Focus group topic guide.**

| **Topic guide** |
| --- |
| **Objectives/areas to explore**   - Explore the HCPs’ views on sibling involvement - HCPs’ attitudes towards documenting sibling involvement - Identify barriers and facilitators regarding sibling involvement, and documentation of sibling involvement - Ideas for improvement |
| **Introduction**   - Name, role in the team, how long they have worked here |
| **Present and discuss audit findings**   - Audit findings: Give everyone the handout, AS presents the key findings and NICE guidelines - Discussion points: Based on the audit results, what are your initial thoughts or reactions? Do these findings align with your experience, or do they surprise you? |
| **Post-it notes exercise: barriers and facilitators of sibling involvement from different viewpoints**   - Benefits of involving siblings   - YP perspective   - Sibling perspective   - Parent perspective   - HCP perspective - What makes it difficult to involve siblings?   - YP perspective   - Sibling perspective   - Parent perspective   - HCP perspective |
| **If not touched upon, areas to explore:**   - What changes (if any) would you like to see in how sibling involvement is documented and recognised in clinical practice? - How could we better integrate sibling involvement into CAMHS ED treatment overall? - Is there anything else about sibling involvement or documentation that we haven’t discussed but you think is important? - What do you think should be the next steps based on our conversation today? |
